# Effects of sustained hyperprolactinemia in late gestation on the mammary parenchymal tissue transcriptome of gilts

**DOI:** 10.1186/s12864-023-09136-4

**Published:** 2023-01-24

**Authors:** Marie-France Palin, Anouk Caron, Chantal Farmer

**Affiliations:** 1 Agriculture and Agri-Food Canada, Sherbrooke R & D Centre, Sherbrooke, QC Canada; 2 Université Laval, Québec, QC Canada

**Keywords:** Domperidone, Gestation, Gilt, Mammary gland, Prolactin, Transcriptomic

## Abstract

**Background:**

Gilts experiencing sustained hyperprolactinemia from d 90 to 109 of gestation showed an early onset of lactogenesis coupled with premature mammary involution. To better understand the molecular mechanisms underlying the premature mammary involution observed in these gilts, a transcriptomic analysis was undertaken. Therefore, this study aimed to explore the effect of hyperprolactinemia on the global transcriptome in the mammary tissue of late gestating gilts and identify the molecular pathways involved in triggering premature mammary involution.

**Methods:**

On d 90 of gestation, gilts received daily injections of (1) canola oil until d 109 ± 1 of gestation (CTL, *n* = 18); (2) domperidone (to induce hyperprolactinemia) until d 96 ± 1 of gestation (T7, *n* = 17) or; (3) domperidone (until d 109 ± 1 of gestation (T20, *n* = 17). Mammary tissue was collected on d 110 of gestation and total RNA was isolated from six CTL and six T20 gilts for microarray analysis. The GeneChip® Porcine Gene 1.0 ST Array was used for hybridization. Functional enrichment analyses were performed to explore the biological significance of differentially expressed genes, using the DAVID bioinformatics resource.

**Results:**

The expression of 335 genes was up-regulated and that of 505 genes down-regulated in the mammary tissue of T20 vs CTL gilts. Biological process GO terms and KEGG pathways enriched in T20 vs CTL gilts reflected the concurrent premature lactogenesis and mammary involution. When looking at individual genes, it appears that mammary cells from T20 gilts can simultaneously upregulate the transcription of milk proteins such as *WAP*, *CSN1S2* and *LALBA,* and genes triggering mammary involution such as *STAT3*, *OSMR* and *IL6R*. The down-regulation of *PRLR* expression and up-regulation of genes known to inactivate the JAK-STAT5 pathway (*CISH*, *PTPN6*) suggest the presence of a negative feedback loop trying to counteract the effects of hyperprolactinemia.

**Conclusions:**

Genes and pathways identified in this study suggest that sustained hyperprolactinemia during late-pregnancy, in the absence of suckling piglets, sends conflicting pro-survival and cell death signals to mammary epithelial cells. Reception of these signals results in a mammary gland that can simultaneously synthesize milk proteins and initiate mammary involution.

**Supplementary Information:**

The online version contains supplementary material available at 10.1186/s12864-023-09136-4.

## Background

Prolactin (PRL) is a regulatory hormone mainly produced and secreted from the anterior pituitary gland. This hormone regulates diverse biological processes and is of particular importance during late gestation and throughout lactation. In swine, inhibition studies clearly demonstrated that PRL is essential for both mammary development and lactation [1, 2]. Administration of recombinant porcine PRL to prepubertal gilts, to maintain PRL concentrations above basal physiological levels, stimulated mammary development [3], whereas exogenous PRL had no effect on mammary development when sows were treated from d 102 of gestation to weaning [4]. However, PRL-treated sows exhibited premature onset of lactogenesis in late gestation but lower milk yield during lactation [4]. By contrast, the study of VanKlompenberg et al. [5] reported that provision of domperidone, a dopamine receptor antagonist known to induce hyperprolactinemia, to late-gestating gilts increased PRL concentrations from days 90 to 97 of gestation and stimulated mammary epithelial cells (MEC) differentiation before farrowing, with a subsequent increase in milk production and piglet growth. In a recent study, we demonstrated that maintaining a sustained hyperprolactinemia throughout the end of gestation in gilts had a negative effect on mammary development [6]. This was evidenced by lower mammary RNA, DNA and protein contents, early involution of mammary parenchymal tissue and lower mRNA abundance of the prolactin receptor (*PRLR*) gene in parenchyma from gilts treated with domperidone for 20 days (T20, from d 90 to 109 of gestation) when compared with controls (CTL) or gilts treated for 7 days (T7, from d 90 to 96) [6]. The T20-treated gilts experienced premature milk synthesis and secretion, which likely led to premature mammary involution in the absence of suckling piglets [6]. Such an effect has been observed by Quaglino et al. [7] who reported that milk stasis can cause mechanical stretch to MECs, which can in turn initiate the activation or inhibition of multiple signaling pathways known to provoke cell death. For example, the janus kinase/signal transducer and activator of transcription (JAK-STAT), nuclear factor-kappa B (NF-κB), phosphatidylinositol 3-kinase/AKT serine/threonine kinase (PI3K-Akt) and the transforming growth factor beta (TGFβ) pathways are known to be involved in mammary involution processes [8, 9]. There is also growing evidence that alternative mechanisms such as the lysosomal-mediated cell death, that occurs independently of executioner caspases (e.g. caspases 3, 6 or 7), can play a role in mammary involution [10]. To better understand the molecular mechanisms underlying the premature mammary involution observed in T20-treated gilts that were exposed to sustained hyperprolactinemia from day 90 to 109 of gestation, a transcriptomic analysis was conducted. Therefore, the present study objectives were to explore the global transcriptomic adaptations that occurred in the mammary tissue of these gilts when compared to that in the CTL treatment, and to identify the molecular pathways involved in triggering premature mammary involution.

## Results

A total of 840 gene transcripts (335 up- and 505 down-regulated) were differentially expressed in mammary parenchymal tissue between T20 and CTL gilts, according to our filter criteria (1.5-fold change and an adjusted *P*-value ≤0.05; Additional file 1: supplementary Table 1). Of these, a total of 283 up- and 364 down-regulated porcine unique genes were identified in T20-treated gilts when compared with CTL animals. Among the list of up-regulated genes, those with the highest fold change (FC) are the whey acid protein (*WAP*; FC: 126.16, *P* = 4.68E-09), alpha(s2)-casein (*CSN1S2*; FC: 81.47, *P* = 0,0001), lactalbumin alpha (*LALBA*; FC: 54.42, *P* = 6.38E-05), transcobalamin 1 (*TCN1*; FC: 35.57, *P* = 2.83E-06) and the acyl-CoA synthetase long chain family member 6 (*ACSL6*; FC: 20.66, *P* = 0002) genes. Genes that were the most down-regulated in T20 vs CTL gilts are the acyloxyacyl hydrolase (*AOAH*; FC: -24.34, *P* = 6.44E-06), chondrolectin (*CHODL*; FC: -20.19, *P* = 2.53E-06), stimulator of chondrogenesis 1 (*SCRG1*; FC: -9.90, *P* = 4.84E-05) and two uncharacterized proteins (C3H2orf40; FC: -9.31, *P* = 0.0001 and LOC106505010; FC: -10.65, *P* = 0.0002).

**Fig. 1:**
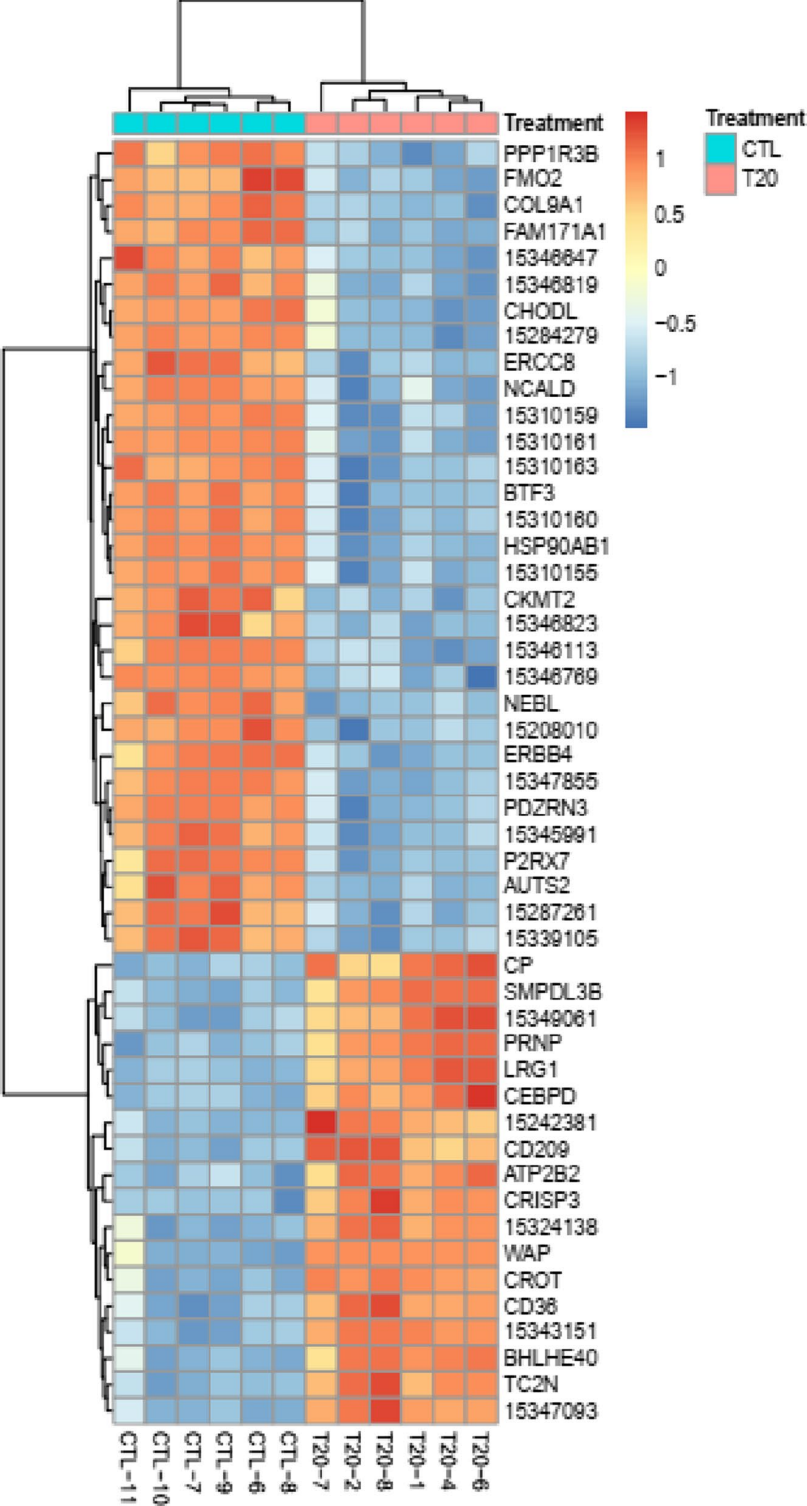
Heatmap with hierarchical clustering of differentially expressed genes between T20 and CTL gilts. Heat map with hierarchical clustering of the top 50 genes with the most significant differences based on their adjusted *P*-values. Each row represents one of the 50 genes and each column one of the 12 samples (6 T20 and 6 CTL gilts) used in microarray analysis. The dendrogram at the top demonstrates similarity among samples, whereas the one on the left shows clusters of genes based on their similar gene expression pattern. Red = positive log fold-change (log FC); Blue = negative log FC

In order to get an overview of differences and similarities in the transcriptional profile of parenchymal tissue samples, a heat map with hierarchical clustering was performed for the top 50 ranked DEGs between T20 and CTL treatments based on their adjusted *P*-values. The heat map dendrogram clearly shows the high similarity of gene expression profiles among samples within the T20 and CTL treatments and a clear difference between treatments (Fig. 1).

### Validation of selected ***differentially expressed genes*** using qPCR analysis

Microarray data were validated by qPCR analysis of 13 up- and 13 down-regulated genes. The whole population of gilts (*n* = 52) was included in this analysis to 1) validate our microarray data (T20 vs CTL gilts) and 2) study the effect of domperidone on the mRNA abundance of these genes in CTL, T7 and T20-treated gilts (Table 1). The qPCR data showed an overall treatment effect (Table 1, *P* <  0.01) for all selected up-regulated genes (atypical chemokine receptor 2 (*ACKR2*), alanyl aminopeptidase (*ANPEP*), basic helix-loop-helix family member E40 (*BHLHE40*), CD14 molecule (C*D14*), CD36 molecule (*CD36*), CAAT enhancer binding protein delta (*CEBPD*), cell death inducing DFFA like effector A (*CIDEA*), extracellular matrix protein 1 (*ECM1*), epidermal growth factor (*EGF*), *LALBA*, leucine rich alpha-2-glycoprotein 1 (*LRG1*), secreted phosphoprotein 1 (*SPP1*) and tandem C2 domains, nuclear (*TC2N*)) and a higher mRNA abundance was found in T20-treated gilts compared with the CTL group (*P* ≤ 0.05), thus confirming our microarray data. Significant differences between T7 and T20 gilts were also observed for all tested up-regulated genes (*P* ≤ 0.05), whereas there was no difference between CTL and T7 gilts. An overall treatment effect (Table 1, *P* <  0.01) was observed for all tested down-regulated genes (calcium/calmodulin dependent protein kinase 1G (*CAMK1G*), *CHODL*, collagen type IX alpha 1 chain (*COL9A1*), C-X-C motif chemokine receptor 4 (*CXCR4*), erb-b2 receptor tyrosine kinase 4 (*ERBB4*), flavin containing monooxygenase 2 (*FMO2*), neurocalcin delta (*NCALD*), purinergic receptor (*P2RX7*), RCC1 and BTBF domain containing protein 1 (*RCBTB1*), regulator of G protein signaling 2 (*RGS2*), *SCRG1*, SHC adaptor protein 4 (*SHC4*) and tudor domain containing 1 (*TDRD1*)) and microarray data were confirmed for all of these genes (T20 > CTL, *P* ≤ 0.05). After 7 days of domperidone treatment (T7), the mRNA abundance of the *CAMK1G*, *COL9A1*, *P2RX7* and *TDRD1* genes was significantly higher than in the CTL group and was lower than the T20 treatment (CTL < T7 < T20, *P* ≤ 0.05). The mRNA abundances of the *CHODL*, *CXCR4*, *ERBB4*, *FMO2*, *NCALD*, *RCBTB1*, *RGS2*, *SCRG1* and *SHC4* genes were similar between the CTL and T7 treatments, whereas they were higher in T20 than in T7 gilts (*P* ≤ 0.05).

**Table 1:** Relative mRNA abundance of selected differentially expressed genes in mammary parenchyma of late-pregnant gilts treated or not with domperidone

Gene symbol	Treatments^1^			*P*-value
	CTL (*n* = 18)	T7 (*n* = 17)	T20 (*n* = 17)	
*Up-regulated genes*				
*ACKR2*	0.19 (0.03)^b^	0.28 (0.04)^b^	3.08 (0.52)^a^	< 0.001
*ANPEP*	0.37 (0.04)^b^	0.34 (0.03)^b^	2.56 (0.45)^a^	< 0.001
*BHLHE40*	0.49 (0.08)^b^	0.46 (0.04)^b^	2.14 (0.20)^a^	< 0.001
*CD14*	0.27 (0.06)^b^	0.38 (0.05)^b^	2.20 (0.27)^a^	< 0.001
*CD36*	0.56 (0.05)^b^	0.45 (0.05)^b^	2.12 (0.28)^a^	< 0.001
*CEBPD*	0.41 (0.06)^b^	0.43 (0.05)^b^	2.32 (0.35)^a^	< 0.001
*CIDEA*	0.13 (0.03)^b^	0.11 (0.02)^b^	3.25 (0.80)^a^	0.003
*ECM1*	0.35 (0.06)^b^	0.37 (0.04)^b^	2.32 (0.23)^a^	< 0.001
*EGF*	0.22 (0.05)^b^	0.26 (0.04)^b^	2.92 (0.39)^a^	< 0.001
*LALBA*	0.12 (0.03)^b^	0.14 (0.03)^b^	2.60 (0.49)^a^	< 0.001
*LRG1*	0.14 (0.03)^b^	0.18 (0.04)^b^	2.52 (0.36)^a^	< 0.001
*SPP1*	0.24 (0.09)^b^	0.33 (0.07)^b^	2.39 (0.44)^a^	< 0.001
*TC2N*	0.54 (0.06)^b^	0.61 (0.05)^b^	1.89 (0.14)^a^	< 0.001
*Down-regulated genes*				
*CAMK1G*	1.64 (0.20)^a^	0.98 (0.10)^b^	0.27 (0.08)^c^	< 0.001
*CHODL*	1.18 (0.13)^a^	1.11 (0.13)^a^	0.21 (0.05)^b^	< 0.001
*COL9A1*	2.29 (0.45)^a^	0.49 (0.14)^b^	0.15 (0.04)^c^	< 0.001
*CXCR4*	0.98 (0.07)^a^	1.08 (0.06)^a^	0.52 (0.06)^b^	< 0.001
*ERBB4*	1.17 (0.14)^a^	0.92 (0.10)^a^	0.54 (0.09)^b^	0.001
*FMO2*	1.34 (0.19)^a^	0.97 (0.18)^a^	0.30 (0.04)^b^	< 0.001
*NCALD*	1.19 (0.07)^a^	1.08 (0.09)^a^	0.56 (0.06)^b^	< 0.001
*P2RX7*	1.41 (0.12)^a^	0.88 (0.11)^b^	0.44 (0.07)^c^	< 0.001
*RCBTB1*	1.27 (0.09)^a^	1.08 (0.09)^a^	0.54 (0.09)^b^	< 0.001
*RGS2*	1.21 (0.14)^a^	1.11 (0.10)^a^	0.44 (0.04)^b^	< 0.001
*SCRG1*	1.37 (0.18)^a^	0.99 (0.09)^a^	0.34 (0.06)^b^	< 0.001
*SHC4*	1.08 (0.09)^a^	1.09 (0.08)^a^	0.73 (0.09)^b^	0.009
*TDRD1*	1.44 (0.14) ^a^	1.05 (0.09)^b^	0.19 (0.05)^c^	< 0.001

^1^CTL = control gilts; T7 = gilts receiving intramuscular injections of 0.5 mg/kg of domperidone from d90 to d 96 of gestation; T20 = gilts receiving intramuscular injections of 0.5 mg/kg of domperidone from d 90 to d 109 of gestation

LSmeans are presented with their individual SEM

^a-c^Values with different superscripts differ at *P* ≤ 0.05

### Functional enrichment analysis of differentially expressed genes

Functional enrichment analyses were carried out to explore the biological significance of identified DEGs between T20 and CTL groups. Table 2 shows the biological process and molecular function GO terms with fold enrichment score (FES) > 1.5 and enrichment *P*-values ≤0.05. A total of 11 biological process GO terms were significantly enriched in T20 compared with CTL gilts and their associated FES ranged from 2.0 to 3.7. Enriched GO terms included the negative regulation of apoptotic process (10 up- and 10 down-regulated genes), metabolic process (7 up- and 4 down-regulated genes), oxidation-reduction process (7 up- and 7 down-regulated genes), positive regulation of I-kappaB kinase/NF-kappaB signaling (7 up- and 4 down-regulated genes), regulation of gene expression (3 up- and 5 down-regulated genes), innate immune response (12 up- and 3 down-regulated genes), positive regulation of NF-kappaB transcription factor activity (4 up- and 5 down-regulated genes), apoptotic process (5 up- and 4 down-regulated genes), inflammatory response (13 up- and 2 down-regulated genes), positive regulation of cell proliferation (4 up- and 11 down-regulated genes) and the response to lipopolysaccharide (5 up- and 3 down-regulated genes). There was a tendency for the negative regulation of apoptotic process GO term after applying a Benjamini-Hochberg correction.

**Table 2:** Over-represented Gene Ontology (GO) terms within the biological process and molecular function categories using differentially-expressed genes in the mammary parenchyma of T20 vs CTL gilts

DAVID (version 6.8)	Count^1^	Gene Symbols^2^	FES^3^	*P*-value^4^
GOTERM - Biological Process – DIRECT				
Negative regulation of apoptotic process GO:0043066	20	*APBB2*, *BIRC5*, *CAT*, ***CITED2***, ***CTSH***, ***DAB2***, ***FOXO1***, ***HSPA5***, *HSP90AB1*, *IGF1R*, ***LTF***, *MYC, NPM1*, ***PIM1***, ***PRNP***, ***PSEN2***, *RARG*, *RPS3A*, *SOX9*, ***STAT3***	2.5	3.5E-4†
Metabolic process GO:0008152	11	*ACSF2*, ***ACSL3***, ***ACSS3***, ***AGPAT3***, ***ARSA***, ***LCLAT1***, ***MAN1A1***, *SCP2*, *SUCLG1*, ***SULF2****, UGT8*	3.4	0.0015
Oxidation-reduction process GO:0055114	14	*AKR1B1*, ***ALDH2***, *CAT*, **CYP4A24**, ***CYP51***, ***DUOX1***, *ETFB*, *FMO2*, ***GPX1***, *HSD17B8*, *MAOB*, *PRDX6*, ***SCD***, ***SOD2***	2.5	0.0035
Positive regulation of I-kappaB kinase/NF-kappaB signaling GO:0043123	11	***CASP1***, *CTNNB1*, ***ECM1***, ***LITAF***, ***LTF***, *MID2*, ***MYD88***, ***NOD1***, ***S100A12***, *S100B*, *TRIM13*	3.0	0.0038
Regulation of gene expression GO:0010468	8	*DNMT3A*, *DNMT3B*, *EIF4A2*, ***FAM46A***, *MEGF8*, ***PHLDA2***, *SHC4*, ***TC2N***	3.7	0.0051
Innate immune response GO:0045087	15	***C3***, ***C9***, ***CD14***, ***CD59***, *HMGB2*, ***JCHAIN***, *MID2*, ***MYD88***, ***PGLYRP1***, ***S100A12***, ***TLR2***, ***TLR4***, ***TMEM173***, ***TNK2,*** *TRIM1*	2.2	0.0080
Positive regulation of NF-kappaB transcription factor activity GO:0051092	9	*CAT*, ***LTF***, *MID2*, ***NOD1***, *NPM1*, *PRDX3*, ***RIPK3***, ***TLR2***, *TRIM13*	3.1	0.0082
Apoptotic process GO:0006915	9	*BIRC5*, ***CASP1***, ***CIDEA****,* ***CTSH***, ***FOXO1****, RPS3,* *SHC4*, *STK26,* ***TMEM173***	2.6	0.0160
Inflammatory response GO:0006954	15	***ACKR2***, *AIMP1*, ***C3***, ***CCL23***, ***CD14***, ***CXCL2***, ***CXCL8***, ***ECM1***, ***MYD88***, ***ODAM***, *P2RX7*, ***PTGER4***, ***S100A12***, ***TLR2***, ***TLR4***	2.0	0.0190
Positive regulation of cell proliferation GO:0008284	15	*CDCA7L*, ***CTSH***, *EMP2*, ***FOLR2***, *MYC,* ***MZB1***, *NPM1*, *NTRK3*, *PDGFC*, *PRDX3*, ***PTPN6***, *RARG*, *SHC4*, *SOX9*, *TGFA*	2.0	0.0190
Response to lipopolysaccharide GO:0032496	8	***CASP1***, ***CXCL2*,** ***CXCL8***, ***CYP27B1***, *HMGB2*, *P2RX7*, *PRDX3*, ***PTGER4***	2.7	0.0270
GOTERM - Molecular Function – DIRECT				
Structural constituent of ribosomes GO: 0003735	18	*MRPL14*, *MRPL46*, *RPL5*, *RPL9*, *RPL10A*, *RPL11*, *RPL22*, *RPL24*, *RPL27*, *RPL31*, *RPL34*, *RPL35A*, *RPL36*, *RPS3*, *RPS3A*, *RPS20*, *SLC25A35*, *SLC25A36*	2.3	0.0019
Poly(A) RNA binding GO:0003723	40	*ALDH6A1*, *BTF3*, *C1H14orf166*, *CDC5L*, ***CSRP1***, *DSP*, *EIF3D*, *EIF3G*, *EIF4A2*, ***FAM46A***, *FTSJ3*, ***GRN***, *HMGN2*, *HNRNPA1*, *HSP90AB1*, *IPO5*, *KHDRBS1*, *LSM3*, *MRPL14*, *NAF1*, *NAP1L1*, *NOP16*, *NPM1*, *NSA2*, *PABPC4*, ***PDIA4***, *PEBP1*, *RAVER2*, *RPL11*, *RPL22*, *RPL27*, *RPL36*, *RPS20*, *RPS3A*, *SNRPD1*, *SNRPG*, *SSRP1*, *SUB1*, *SUCLG1, TRAP1*	1.5	0.0120
Chromatin DNA binding GO:0031490	6	***EZH2***, *GRHL3*, *PPARGC1A*, ***STAT3***, *THRB*, *WBP2*	4.1	0.0150
RNA binding GO:0003723	15	*CIRBP*, *LSM3*, *MRPL39*, *PPARGC1A*, *RPL9*, *RPL10A*, *RPL22*, *RPL34*, *RPS3*, *RPS20*, *SMN1*, *SNRPD1*, *SNRPE*, *SNRPF*, *UBR5*	2.0	0.0210
Zinc ion binding GO:0008270	43	*ADAMTS6*, *ADAMTS9*, ***ANPEP***, *BIRC5*, ***BMP1***, ***CPM***, ***CSRP1***, *CSRP2*, *DMD*, *DTNB*, *DTX4*, *KAT7*, *LIMK2*, *LTA4H*, ***MAN2A1***, *MID2*, *MMP16*, ***MT-2B***, *MYLIP*, *NRAP*, ***PAM***, *PAPLN*, *PDLIM1*, ***PGLYRP1***, *PHC1*, *RARG*, *RNF113A*, ***RNF125***, *RNF144B*, *RUFY1*, ***S100A12***, *S100B*, ***SEC24A***, *TET1*, *THRB*, ***TRAF7***, *TRIM13*, *UBR5*, ***VDR***, *ZCCHC11*, ***ZDHHC2***, *ZMIZ1*, *ZNRF3*	1.3	0.0480

^1^Count = number of genes

^2^Bold = up-regulated genes and Plain text = down-regulated genes in T20 compared with CTL gilts; Underlined = genes that were validated with qPCR analysis

^3^FES = Fold Enrichment Score

^4^Gene ontology term enrichment *P-*values; †, tendency after Benjamini-Hochberg correction

For the molecular function GO terms, the following terms were significantly enriched: structural constituent of ribosomes (18 down-regulated genes), poly(A) RNA binding (4 up- and 36 down-regulated genes), chromatin DNA binding (2 up- and 4 down-regulated genes), RNA binding (15 down-regulated genes) and zinc ion binding (14 up- and 29 down-regulated genes) (Table 2). None of these terms remained significant after the Benjamini-Hochberg correction. Associated FES ranged from 1.3 to 4.1.

### KEGG pathways

The list of up- and down-regulated genes (Additional file 2: supplementary Table 2) was submitted to KEGG pathway analysis to identify over-represented pathways. Pathways having more than 5 genes and an EASE thresholds ≤0.05 are shown in Table 3. This analysis revealed a total of 23 pathways that were enriched in T20 compared to CTL gilts. Among these, the following KEGG pathways were not considered as they are not biologically relevant for the current study: prostate cancer, legionellose, pertussis, biosynthesis of antibiotics, leishmaniasis, arrhythmogenic right ventricular cardiomyopathy, hematopoietic cell lineage and measles. Among the 15 remaining KEGG pathways (Table 3), two were significant after applying a Benjamini-Hochberg correction (metabolic pathways, *P* = 0.0041; JAK-STAT signaling pathway, *P* = 0.082 tendency). Associated FES for the 15 enriched KEGG pathways ranged from 1.6 to 3.6.

**Table 3:** KEGG molecular pathways enriched using differentially expressed genes in the mammary parenchyma of T20 vs CTL gilts

KEGG Pathways	Count^1^	Gene Symbols^2^	FES^3^	*P*-value^4^
Metabolic pathways	77	*ACAT1,* ***ACSL3****,* ***ACSL6****,* ***ACSS3****, ADSSL1,* ***AGPAT3****, AKR1B1,* ***ALDH1B1****, ALDH2, ALDH6A1,* ***AMPD3****, AMT,* ***ANPEP****, AOX1, ATIC, ATP6V1D, B3GALT2, B3GALT5,* ***BDH1****, C1GALT1, CDS1,* ***CHDH****, CKMT2, CRLS1,* ***CYP4A24****,* ***CYP27A1****,* ***CYP27B1****,* ***CYP51****, DGUOK, DNMT3A, DNMT3B, FAH, FPGS,* ***GALNT3****,* ***GALNT15****,****GCLC****,****GFPT1****,HIBCH, HPRT1,* ***HSD17B7****,HSD17B8, IMPDH2,* ***LALBA****,* ***LCLAT1****,* ***LIPG****, LIPT1,* ***LPIN2****,* ***LSS****, LTA4H,* ***MAN1A1****,* ***MAN1A2****,* ***MAN1C1****,* ***MAN2A1****, MAOB,* ***MOCS1****, MTMR2,* ***NT5E****, OCRL,* ***PAPSS2****, PIK3C3,* ***PLB1****, PLCH1, PMM1, PRDX6, PSAT1, SCP2,* ***SGMS2****,* ***SHMT2****,* ***SRM, ST3GAL1****, ST3GAL5,* ***ST6GAL1****, ST6GAL2, SUCLG1, UGT8, UXS1,* ***XDH***	1.6	1.7E-5**
Jak-STAT signaling pathway	16	*CCND1,* ***CCND2****,* ***CISH****,* ***CSF2RA****,* ***GHR****, IFN-ALPHA-9,* ***IFNGR2****,* ***IL2RG****,* ***IL6R****,* ***IL13RA1****, MYC,* ***OSMR****,* ***PIM1****, PRLR,* ***PTPN6****,* ***STAT3***	2.7	6.7E-4†
PI3K-Akt signaling pathway	26	*CCND1,* ***CCND2,*** *CHRM1,* ***CREB3L1****,* ***EGF****,* ***GHR****,* ***GNB4****, GNG2, HSP90AB1, IFN-ALPHA-9, IGF1R,* ***IL2RG****,* ***IL6R, ITGA2****,* ***ITGB6****, KITLG,* ***MCL1****, MYB, MYC,* ***OSMR****, PDGFC,* ***PPP2R3A****, PRLR,* ***SPP1****,* ***TLR2****,* ***TLR4***	1.9	2.1E-3
Ribosome	15	*MRPL14, RPL5, RPL9, RPL10A, RPL11, RPL22, RPL24, RPL27, RPL31, RPL34, RPL35A, RPL36, RPS3, RPS3A, RPS20*	2.5	2.2E-3
PPAR signaling pathway	9	***ACSL3****,* ***ACSL6****,* ***ANGPTL4****,* ***CD36****,* ***CYP4A24****,* ***CYP27A1****, DBI,* ***SCD****, SCP2*	3.2	6.3E-3
Purine metabolism	15	***ADCY1****, ADSSL1,* ***AMPD3****, ATIC, DGUOK, GUCY1A3, HPRT1, IMPDH2,* ***NT5E****, NUDT5,* ***PAPSS2****, PDE4D, PDE6D, PDE9A,* ***XDH***	2.0	1.7E-2
Prolactin signaling pathway	8	*CCND1,* ***CCND2****,* ***CISH****,* ***FOS****, PRLR,* *SHC4,* ***STAT3****,* ***WAP***	2.9	1.9E-2
Fatty acid degradation	6	*ACAT1,* ***ACSL3****,* ***ACSL6****,* ***ALDH1B1***, *ALDH2*, ***CYP4A24***	3.6	2.4E-2
Tryptophan metabolism	6	*ACAT1,* ***ALDH1B1***, *ALDH2*, *AOX1*, *CAT*, *MAOB*	3.6	2.4E-2
Thyroid hormone synthesis	8	***ADCY1****,* ***ATP1B2****,* ***CREB3L1****,* ***FXYD2****,* ***GPX1****,* ***HSPA5****,* ***PDIA4****, SLC5A5*	2.7	2.7E-2
Glycerolipid metabolism	7	***AGPAT3****, AKR1B1*, ***ALDH1B1***, *ALDH2*, ***LCLAT1***, ***LIPG***, ***LPIN2***	2.9	3.0E-2
Fatty acid metabolism	6	*ACAT1,* ***ACSL3****,* ***ACSL6****,* ***ELOVL5****, HACD3,* ***SCD***	3.2	3.8E-1
Valine, leucine and isoleucine degradation	6	*ACAT1,* ***ALDH1B1***, *ALDH2*, *ALDH6A1*, *AOX1, HIBCH*	3.0	4.8E-2
Protein processing in endoplasmic reticulum	13	***DERL3***, ***DNAJB11***, ***EDEM1***, *HSP90AB1*, ***HSPA5****,* ***HYOU1***, ***MAN1A1***, ***MAN1A2***, ***MAN1C1***, ***PDIA4***, ***SEC24A***, *SEC61A2*, ***TXNDC5***	2.0	3.3E-2
Phagosome	12	*ATP6V1D*, ***C3*,** ***CD14***, ***CD36***, ***CD209***, *FCGR2B*, ***ITGA2***, *PIK3C3*, *SEC61A2*, ***TLR2***, ***TLR4***, ***TUBA1C***	2.0	3.5E-2

^1^ Count = number of genes

^2^ Bold = up-regulated genes and Plain text = down-regulated genes in T20 compared with CTL gilts; Underlined = genes that were validated with qPCR

^3^ FES = Fold Enrichment Score

^4 ^** *P* < 0.01 and † tendency after Benjamini-Hochberg correction

### STRING interaction network

**Fig. 2:**
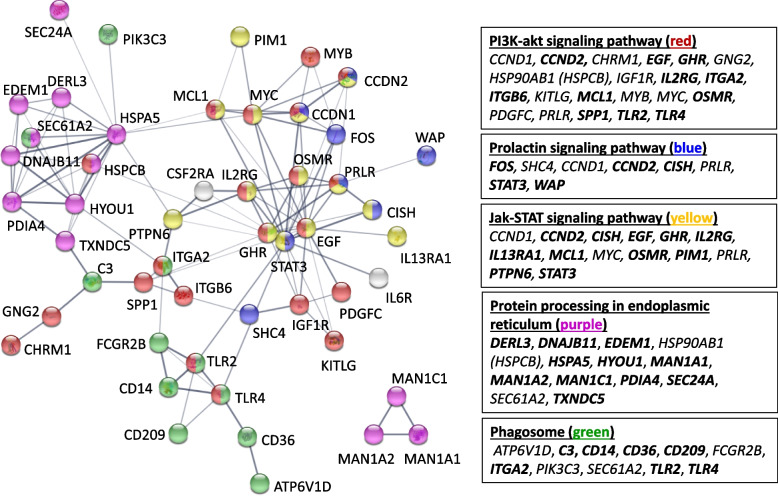
STRING-generated interaction network among selected enriched KEGG pathways. The network image shows interactions between proteins encoded by DEGs identified in the following KEGG molecular pathways: PI3K-Akt signaling pathway (ssc04151), prolactin signaling pathway (ssc04917), JAK-STAT signaling pathway (ssc04630), protein processing in endoplasmic reticulum (ssc04141) and phagosome (ssc04145). Clusters of genes/proteins within specific KEGG pathways are indicated in boxes and by corresponding colored nodes in the network image. Bold = up-regulated genes; Plain text = down-regulated genes in T20 vs CTL gilts. The thickness of the lines represents the confidence prediction of the interaction between 2 genes (thinnest: confidence 0.400; medium: confidence 0.700; thickest: confidence 0.900). Nodes with no interaction with other nodes were deleted. The interaction score was set at > 0.400 and KEGG pathway enrichment significance at *P* > 0.05 (FDR Benjamini-Hochberg)

The STRING database was used to look for interconnections between enriched KEGG pathways and also between DEGs. For this analysis, the downloaded gene list included genes within the following KEGG pathways: PI3K-Akt signaling pathway (ssc04151), prolactin signaling pathway (ssc04917), JAK-STAT signaling pathway (ssc04630), protein processing in endoplasmic reticulum (ssc04141) and phagosome (ssc04145). These pathways were chosen because of their known or suspected roles during mammary development and involution [8, 9]. This analysis generated an interaction network consisting of 53 nodes (genes/proteins) and 116 edges (Fig. 2). Three nodes are not shown in the network because they had no interaction with any other gene. Genes with the highest number of interactions with other genes/proteins are *STAT3* (18 edges with cyclin D1 (*CCND1*), cyclin D2 (*CCND2*), cytokine inducible SH2 containing protein (*CISH*), colony stimulating factor 2 receptor subunit alpha (*CSF2RA*), *EGF*, Fos proto-oncogene, AP1 transcription factor subunit (*FOS*), growth hormone receptor (*GHR*), heat shock protein 90 alpha family class B member 1 (*HSP90AB1*), insulin-like growth factor-1 receptor (*IGF1R*), interleukin 2 receptor subunit gamma (*IL2RG*), interleukin 6 receptor (*IL6R*), kit ligand (*KITLG*), MCL1 apoptosis regulator, BCL2 family member (*MCL1*), MYC proto-oncogene, BHLH transcription factor (*MYC*), oncostatin M receptor (*OSMR*), protein tyrosine phosphatase non-receptor type 6 (*PTPN6*), toll like receptor 2 (*TLR2*) and toll like receptor 4 (*TLR4*)), *EGF* (14 edges with *CCND1*, *FOS*, *GHR*, *IGF1R*, *IL2RG*, interleukin 13 receptor subunit alpha 1 (*IL13RA1*), *KITLG*, *MCL1*, *MYC*, *OSMR*, *PRLR*, *STAT3*, platelet derived growth factor C (*PDGFC*) and *SPP1*)) and heat shock protein family A (HSP70) member 5 (*HSPA5*) (13 edges with derlin 3 (*DERL3*), DnaJ heat shock protein family (Hsp40) member B11 (*DNAJB11*), ER degradation enhancing alpha-mannosidase like protein 1 (*EDEM1*), *HSP90AB1*, hypoxia up-regulated 1 (*HYOU1*), *MCL1*, *MYC*, protein disulfide isomerase family A member 4 (*PDIA4*), phosphatidylinositol 3-kinase catalytic subunit type 3 (*PIK3C3*), *PTPN6*, SEC24 homolog A, COPII coat complex component (*SEC24A*), SEC61 translocon subunit alpha 2 (*SEC61A2*) and thioredoxin domain containing 5 (*TXNDC5*)). Enrichment significance (FDR) was 4.66E-25 for the PI3K-Akt signaling pathway, 1.29E-10 for the prolactin signaling pathway, 3.23E-17 for the JAK-STAT signaling pathway, 3.78E-15 for the protein processing in endoplasmic reticulum and 8.82E-13 for phagosome KEGG pathways.

## Discussion

Current results help to better understand the molecular mechanisms underlying the premature mammary involution observed in gilts after 20 days of sustained hyperprolactinemia in late pregnancy. Treating gilts with domperidone from d 90 to 109 of gestation resulted in important transcriptional adaptations compared with control animals. This is evidenced by the large number of gene transcripts (335 up- and 505 down-regulated) identified in T20 vs control gilts and by the heat map dendrogram showing clear differences in expression profiles between treatments (T20 vs CTL). For the qPCR analysis of selected DEGs in mammary parenchymal tissue, maintaining high circulating concentrations of prolactin for 20 days provoked more alterations in mRNA abundance (T20 vs CTL) compared with a shorter treatment period (T7 vs CTL). The limited effect of domperidone after 7 days of treatment was expected since T7 and CTL gilts had similar mammary composition variables [6]. On the other hand, the important downregulation (4 to 15 fold changes) of *CAMK1G*, *COL9A1*, *P2RX7* and *TDRD1* mRNA abundance (this study) and the higher parenchyma involution score in T7 than CTL gilts [6] suggest that the involution process is already initiated after 7 days of domperidone treatment.

Among the up-regulated genes, those with the highest fold-change between T20 and CTL gilts include three major milk proteins (*WAP*, *CSN1S2*, *LALBA*) known to be up-regulated during lactogenesis [11], a long-chain acyl-CoA synthase 6 (*ACSL6*) involved in milk triglycerides biosynthesis [12] and *TCN1*, a vitamin B-12 binding protein found in breast milk [13]. These results indicate that gilts treated with domperidone for 20 days experienced a premature onset of lactogenesis, a process also observed in Caron et al. [6] whereby T20 gilts had abundant milk secretion on d 110 of gestation. Among the most down-regulated DEGs in T20 vs CTL gilts, a cytokine-like protein (*SCRG1*) and a c-type lectin named chondrolectin (*CHODL*) were identified. Overexpression of *CHODL* has been reported to increase cell migration and invasion and to promote growth in a number of mammalian cell lines [14, 15], while a reduction of its expression suppresses cellular growth [15]. The expression of *SCRG1* decreases during differentiation of human mesenchymal stem cells (hMSC) and recombinant human SCRG1 suppresses differentiation of hMSC while preserving its cellular self-renewal and migration ability [16]. The role of SCRG1 and CHOLD in mammary involution has never been investigated, but the presence of alveolar progenitor cells in the involuted mammary gland [17] and their lower expression in T20 gilts suggest a possible role of these genes in the extensive cellular remodeling occurring during the involution process. The *AOAH* transcript, which was also identified among the top five down-regulated genes, encodes for a lipase that removes fatty acyl chains from lipopolysaccharides (LPS) [18], thus preventing damaging inflammatory responses to gram-negative bacteria. The observed reduction in *AOAH* gene expression may therefore predispose T20 gilts to mammary inflammation. Of interest, the inflammatory response and the response to lipopolysaccharide GO terms were over-represented in T20 gilts and included up-regulated genes such as the LPS activated receptors *TLR2* and *TLR4*, known to induce an inflammatory response, and TLRs signal transducer adaptor (MYD88 innate immune signal transduction adaptor (*MYD88*)), co-receptor (*CD14*) and downstream inflammatory chemokines (C-C motif chemokine ligand 23 (*CCL23*), C-X-C motif chemokine ligand 2 (*CXCL2*), C-X-C motif chemokine ligand 8 (*CXCL8*)) [19]. On the other hand, the upregulation of the TLR4 endogenous ligand (S100 calcium binding protein A12 (*S100A12*)) [20] in T20 gilts may also suggest that an LPS-independent mechanism has been activated to regulate the inflammatory response.

Among the molecular function GO terms that were enriched in T20 gilts, the structural constituent of ribosomes, Poly(A) RNA binding and RNA binding terms are of particular interest. Genes within these terms include several ribosomal protein components of the 60S (*RPL5*, *RPL9*, *RPL10A*, *RPL11*, *RPL22*, *RPL24*, *RPL27*, *RPL31*, *RPL34*, *RPL35A*, *RPL36*), 40S (*RPS3*, *RPS3A*, *RPS20*) and mitochondrial 39S (*MRPL14*, *MRPL39*, *MRPL46*) subunits that were all down-regulated in T20 vs CTL gilts. A lower expression of nuclophosmin 1 (*NPM1*), known to be involved in several stages of ribosome biogenesis [21], was also observed. This may suggest a general decrease in ribosomal biogenesis and an associated reduction in protein translation. Such a hypothesis is supported by the observed decrease in total protein in mammary parenchyma of T20 gilts compared with CTL gilts [6]. It is well established that the MYC proto-oncogene regulates the expression of several ribosomal proteins, as well as other factors needed for ribosome biogenesis and initiation of translation [22]. Interestingly, NPM1 was identified as a MYC-activated target [23]. Therefore, the downregulation of *MYC*, *NPM1* and the translation initiation factors *EIF3D*, *EIF3G* and *EIF4A2* in T20 gilts may explain, at least in part, the observed reduction in protein synthesis and ribosomal proteins gene expression.

Under the molecular function GO terms, the zinc ion binding term was enriched in T20 gilts. As a structural component of many enzymes and transcription factors, the zinc ion can affect different molecular processes during mammary development, lactation and mammary involution [24]. For instance, McCormick et al. [24] have reported an accumulation of zinc in lysomomes of the involuting mouse mammary glands, thus suggesting an important role during the lysosomal-mediated cell death occurring in the first phase of involution. Among the 43 genes identified in this GO term, 9 are E3 ubiquitin protein ligases (*DTX4*, *MYLIP*, *RNF113A*, *RNF125*, *RNF144B*, *TRAF7*, *TRIM13*, *UBR5*, *ZNRF3*) and 8 are hydrolytic enzymes (*ADAMTS6*, *ADAMTS9*, *ANPEP*, *BMP1*, *CPM*, *LTA4H*, *MMP16*, *PAPLN*). E3 ligases selectively modify target proteins with ubiquitin tags, a modification leading to proteasomal degradation. Protein ubiquitination has also emerged as an important regulator of protein activity and trafficking, thus controlling a wide array of cellular processes [25]. The contribution of hydrolytic enzymes is essential for the remodeling of the involuting mammary gland and the modulation of four metalloproteases (*BMP1*, *ADAMTS6*, *ADAMTS9*, *MMP16*), involved in the extracellular matrix (ECM) degradation [26], and four peptidases (*ANPEP*, *CPM*, *LTA4H*, *PAPLN*) in T20 gilts suggests that sustained hyperprolactinemia in late gestation, in the absence of suckling piglets, can trigger proteolytic events of the involution program.

The protein-protein interaction network generated with the STRING tool clearly demonstrated that the JAK-STAT, PI3K-Akt and prolactin signaling pathways, as well as the protein processing in endoplasmic reticulum and phagosome KEGG pathways are all interconnected. With this analysis, *HSPA5*, *EGF* and *STAT3* were identified as having the highest number of interactions with other DEGs. HSPA5 is a member of the Hsp70 chaperone family found in endoplasmic reticulum (ER) and is known to facilitate protein folding and to direct unfolded or misfolded proteins to the ER for degradation. HSPA5 also regulates intracellular calcium homeostasis [27], it is a key up-regulated transcript during regenerative involution in the bovine mammary gland [28] and a PRL-response protein in a mouse MEC line [29]. Interestingly, co-immunoprecipitation experiments have shown that HSPA5 is part of an ER multi-protein complex that includes HSP90AB1, DNAJB11, HYOU1 and PDIA4 [30, 31], and these were also up-regulated in T20 gilts (except for *HSP90AB1*) and were identified under the over-represented protein processing in ER KEGG pathway. T20 gilts experienced premature milk synthesis that, in the absence of suckling piglets, led to the accumulation of calcium and milk proteins such as *WAP*, *CSN1S2* and *LALBA*. Such accumulation increases cellular stress and triggers mammary involution. This can also initiate the unfolded protein response (UPR) known to increase the expression of ER-resident chaperones such as HSPA5 [32].

A 13.3-fold increase in *EGF* mRNA abundance was observed in the parenchymal tissue of T20 gilts compared with the CTL group. A 100-fold increase in *EGF* transcript abundance has been observed in mice mammary glands at the onset of lactation and inhibition of *EGF* impaired lactation [33]. Variations in EGF concentrations can lead to different cell fates, with low concentrations (0,16 nM) promoting cell growth, and high concentrations (16 nM) inducing apoptosis through the activation of STAT3 [34]. Interestingly, EGF is one of the most abundant growth factor in sow colostrum and milk [35] and, in the absence of suckling piglets, T20 gilts may accumulate EGF in milk secretions, which would in turn activate STAT3 and initiate the involution process.

Among the DEGs showing interactions with *STAT3* in the STRING analysis, we observed known STAT3 downstream targets such as *CCND1*, *FOS*, *HSP90AB1*, *IL6R*, *MCL1*, *MYC* and *OSMR* [36, 37], genes known to activate STAT3 (*EGF*, *IL6R*, *OSMR*, *TLR2*, *TLR4*) [34, 38, 39] and to inhibit STAT5 trans-activation (*CISH*) [40]. STAT3 is a transcription factor having a pivotal role in the regulation of cell death and the remodeling in mammary involution [41]. Cessation of the suckling stimulus was reported to activate STAT3, with LIF (leukemia inhibitory factor) being the initial activating factor, followed by oncostatin M and its receptor (OSMR) during the second phase of involution [39, 42]. The upregulation of genes activating STAT3 and inhibiting STAT5 activation, and the up-regulation of several STAT3 downstream targets, strongly suggest that the premature mammary involution observed in T20 gilts is mediated through the activation of STAT3. On the other hand, the sustained hyperprolactinemia, the presence of milk secretions and the high transcript abundance of milk proteins also suggest an activation of STAT5 in T20 gilts. Indeed, PRL binding to PRLR activates the JAK2-STAT5 signaling pathway, with downstream effects on MEC survival and milk synthesis [43]. Therefore, the circulating PRL concentrations in T20 gilts were high enough to sustain milk secretion, but insufficient to prevent mammary involution in the absence of suckling piglets.

The prolactin, JAK-STAT and PI3K-Akt signaling pathways were all enriched KEGG pathways in T20 gilts. The PI3K-Akt survival axis is activated by lactogenic hormones such as PRL and insulin-like growth factors and is down-regulated with initiation of mammary involution [8]. When looking at the genes within these pathways, up-regulated genes included *STAT3*, genes activating STAT3 (*EGF*, *IL6R*, *OSMR*, *TLR2*, *TLR4*) [34, 38, 39] or known to disrupt JAK2/STAT5 signaling (*PTPN6/SHP1*, *CISH*) [44] and cytokine receptors regulating the immune and inflammatory responses (*CSF2RA*, *IL2RG*, *IL13RA1*, interferon gamma receptor 2 (*IFNGR2*)) [45]. Down-regulated transcripts included genes having key roles in cell cycle/proliferation such as *CCND1*, *MYC* and the MYB proto-oncogene (*MYB*) [46]. Other genes with important roles in cell survival (*IGF1R*, *KITLG/SCF*, *PDGFC*, *PRLR*, *SHC4/SHCD*) were also down-regulated in T20 gilts. The reduction in *PRLR* transcript abundance in T20 gilts may affect the PRLR-JAK2/STAT5 intracellular signaling and initiate mammary involution, as previously demonstrated with neutralizing PRLR antibodies [47]. The positive regulation of cell proliferation GO terms was also over-represented in T20 gilts and included down-regulated genes that positively regulate cell proliferation and differentiation (cell division cycle associated 7 like (*CDCA7L*), epithelial membrane protein 2 (*EMP2*), *MYC*, *NPM1*, neurotrophic receptor tyrosine kinase 3 (*NTRK3*), platelet derived growth factor C (*PDGFC*), peroxiredoxin 3 (*PRDX3*), retinoic acid receptor gamma (*RARG*), *SHC4*, SRY-Box transcription factor 9 (*SOX9*), transforming growth factor alpha (*TGFA*)). Taken together, these results clearly reflect a downregulation of cellular proliferation and differentiation, likely mediated through the activation of STAT3 and disruption of JAK2/STAT5 signaling [47, 48].

Although the role of STAT3 in initiating the involution process is well characterized, the activation of STAT3 alone is insufficient to trigger involution in the absence of NF-κB [49]. Proposed mechanisms for the activation of NF-κB during the first phase of involution include milk stasis and a decline in circulating lactogenic hormones, whereas loss of cell-cell or cell-matrix contacts have been suggested in the second phase [50]. In this study, the positive regulation of I-kappaB kinase/NF-kappaB signaling and the positive regulation of NF-kappaB transcription factor activity GO terms were over-represented in T20 gilts. Several genes within these two GO terms are involved in the TLRs-NF-κB signaling pathway, including the *TLR2* receptor and its *MYD88* adaptor [19], TLRs endogenous ligands (*S100A12*, lactotransferrin (*LTF*)) [20, 51], the E3 ubiquitin ligase tripartite motif containing 13 (*TRIM13*) [52] and a MYD88 downstream partner (LPS induced TNF factor (*LITAF*)) known to up-regulate inflammatory cytokines [53]. The *ECM1*, midline 2 (*MID2*), *MYD88*, nucleotide binding oligomerization domain containing 1 (*NOD1*), *S100A12*, *TLR2* and *TRIM13* genes are all known NF-κB activators [54, 55]. Activation of the TLR-NF-κB signaling pathway in turn induces the production of inflammatory cytokines and chemokines such as interleukin 2 (IL2), interleukin 6 (IL6), tumor necrosis factor (TNF), CXCL8 and CXCL2, and the activation of the inflammatory caspase 1 (CASP1) [56]. The *CXCL2* and *CXCL8* chemokines, *CASP1* and IL6, IL2 and TNF receptors (*IL6R*, *IL2RG*, TNF receptor superfamily member 17 (*TNFRSF17*)) transcripts were all up-regulated in T20 gilts, thus suggesting an activation of NF-κB. In early involution, TLRs activation in nonprofessional phagocytic MEC induces the secretion of CXCL8, which then recruits neutrophils and, eventually, recruits professional phagocytes (macrophages) [57]. Phagocytic MEC and macrophages are crucial for the clearance of dead cells, cell debris and milk components during involution.

In the first phase of mammary involution, the programmed cell death relies on a lysosomal-mediated pathway that requires STAT3 activation and the release of cathepsins in the cytosol where they act as executioner proteases [10]. The up-regulation of *STAT3* and cathepsins (*CTSH*, *CTSC*) transcripts abundance, and the increase of *LTF*, stimulator of interferon response CGAMP interactor 1 (STING1/*TMEM173*) and *CASP1*, all known to be involved in lysosomal-mediated cell death [58, 59], suggest that this pathway is well engaged in T20 gilts. Current findings identified these genes within the negative regulation of the apoptotic process and the apoptotic process GO terms. The transcription factor forkhead box O1 (*FOXO1*) and its downstream target *CIDEA* were also up-regulated in T20 gilts. In their unphosphorylated forms, FOXOs proteins are known regulators of cell death, as demonstrated by their capacity to increase the expression of pro-apoptotic factors such as BCL2 like 11 (*BCL2L11*/*BIM*) and the fas ligand (*FASLG*/*FASL*) [60], and *CIDEA*, a factor that can induce DNA fragmentation and free fatty acid-mediated apoptosis [61]. Within the same GO terms, three anti-apoptotic genes (Cbp/P300-interacting transactivator 2 (*CITED2*), *HSPA5*, pim-1 proto-oncogene (*PIM1*)) were up-regulated in T20 gilts. This increase may be explained by the observed hyperprolactinemia since prolactin and STAT5 are known transcriptional activators of *CITED2* [62], *HSPA5* [63] and *PIM1* [64]. The up-regulation of both pro- and anti-apoptotic genes in T20 gilts may be explained by the presence of simultaneous hyperprolactinemia and milk stasis, which may activate conflicting pro-survival and cell death signals in the mammary gland. Similarly, down-regulated genes within these two GO terms included both activators of apoptosis such as *MYC*, nucleophosmin 1 (*NMP1*) and serine/threonine kinase 26 (*STK26*) [65–67] and survival factors such as the baculovirus IAP repeat containing 5 (*BIRC5/survivin*)*,* ribosomal protein S3 (*RPS3*) and ribosomal protein S3A (*RPS3A*) [68–70].

During the second irreversible phase of programmed cell death, adipocytes regenerate and rapidly become visible [71]. Histological analyses of the mammary parenchyma revealed a greater accumulation of immune cells in T20 than in CTL and T7 gilts, but large clusters of adipocytes were not detected [6], possibly due to the sustained hyperprolactinemia, which may prevent completion of the involution program. On the other hand, the identification of DEGs and enriched pathways involved in fatty acid (FA) metabolism (PPAR signaling, FA degradation, FA metabolism, Glycerolipid metabolism) in T20 gilts may suggest an initiation of adipocytes repopulation or an increase in milk fat synthesis. The up-regulation of genes involved in milk FA synthesis (acyl-CoA synthetase long chain family member 3 (*ACSL3*), *ACSL6*, centaurin-gamma-3 (*AGAP3*), *CD36*, ELOVL fatty acid elongase 5 (*ELOVL5*), lipin 2 *(LPIN2)* and stearoyl-CoA desaturase (*SCD*)) [72] in T20 gilts indeed suggests an increase in milk fat synthesis.

## Conclusion

**Fig. 3:**
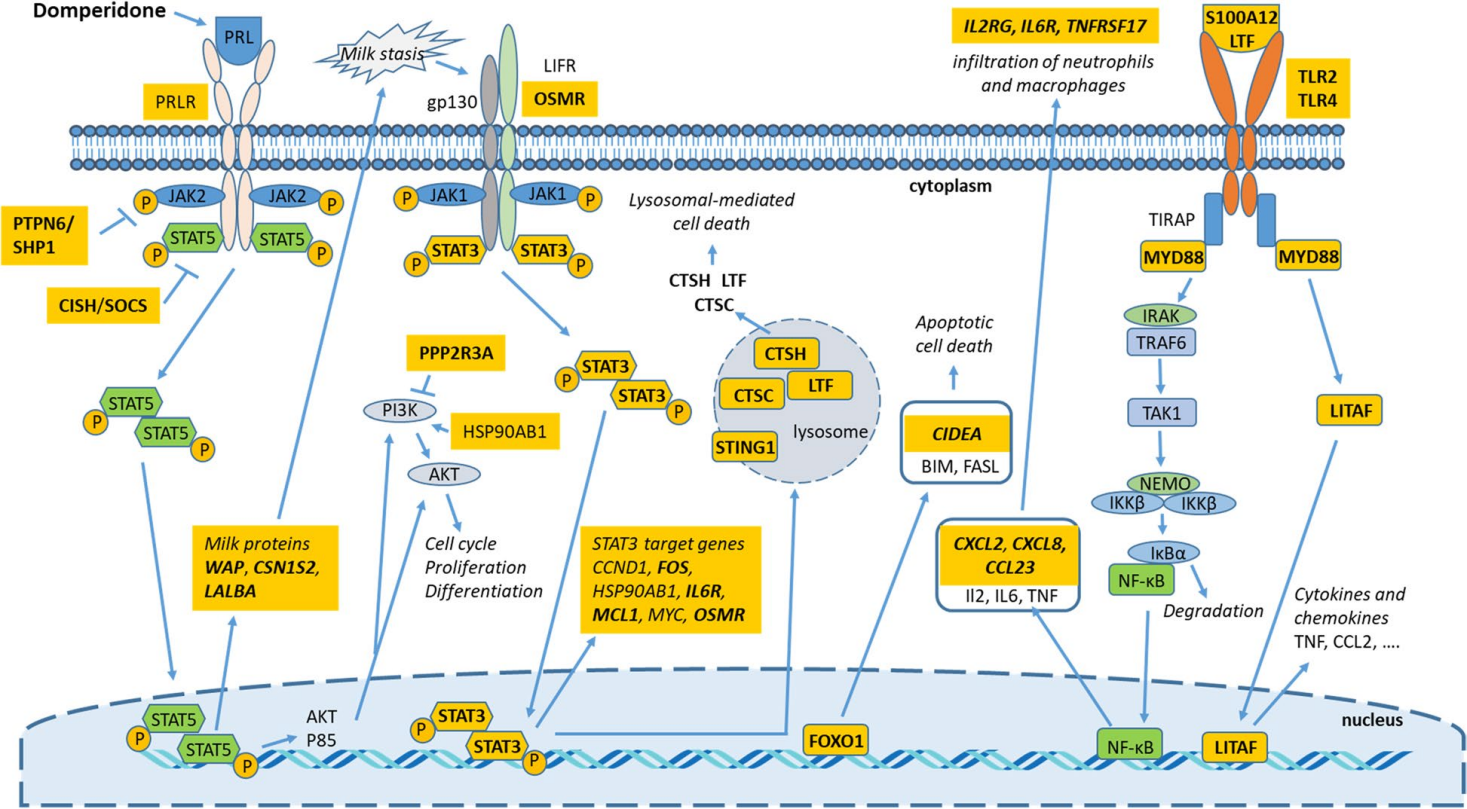
Schematic representation of transcriptomic adaptations and molecular pathways involved in triggering premature mammary involution in gilts that experienced sustained hyperprolactinemia from d 90 to 109 of gestation. Injections of the dopamine receptor antagonist domperidone from d 90 to 109 of gestation provoked sustained hyperprolactinemia. Upon binding to its receptor (PRLR), PRL activates the JAK2-STAT5 signaling pathway, which then induces the transcription of milk proteins such as *WAP*, *CSN1S2* and *LALBA*. STAT5 can also induce the transcription of *AKT* and *P85*, two proteins that are part of the PI3k-AKT signaling pathway. In the absence of milk removal, the JAK1-STAT3 signaling pathway is activated leading to programmed cell death. In the first phase of mammary involution, the lysosomal cell death is mediated through the LIFR-JAK1-STAT3 signaling pathway. In the second phase, oncostatin M and its receptor (OSMR) mediate the apoptotic cell death. The up-regulation of *FOXO1* is known to induce cellular apoptosis. The downregulation of *PRLR* transcript and the increased expression of *PTPN6* and *CISH* suggest a negative feedback loop to reduce the activation of the PRLR-JAK2-STAT5 signaling pathway. The upregulation of *PPP2R3A* and downregulation of *HSP90AB1* may inactivate the PI3K-Akt signaling pathway. The up-regulation of *S100A12*, *LTF*, *TLR2*, *TLR4*, *MYD88*, *CXCL2*, *CXCL8* and *CCL23* transcripts suggests an activation of the TLR-NF-κB signaling pathway. The up-regulation of *CXCL2*, *CXCL8* and *CCL23* chemokines, as well as the *IL2*, *IL6* and *TNF* receptors may induce the infiltration of neutrophils and professional phagocytes (macrophages) in the involuting mammary glands. Differentially expressed genes are indicated in yellow boxes with up-regulated genes in bold character and down-regulated genes in plain text. *AKT*: AKT serine/threonine kinase; *BIM*/*BCL2L11*: BCL2 like 11; *CCL2*: C-C motif chemokine ligand 2; *CCND1*: cyclin D1; *CIDEA*: cell death inducing DFFA like effector A; *CISH*: cytokine inducible SH2 containing protein; *CSN1S2*: alpha(s2)-casein; gp130: glycoprotein 130; *CTSC*: cathepsin C; *CTSH*: cathepsin H; *CXCL2*: C-X-C motif chemokine ligand 2; *CXCL8*: C-X-C motif chemokine ligand 8; *FASL*: fas ligand; *FOS*: Fos proto-oncogene, AP1 transcription factor subunit; *FOXO1*: forkhead box O1; *HSP90AB1*: heat shock protein 90 alpha family class B member 1; *IKBα*: NFKB inhibitor alpha; *IKKβ*: inhibitor of nuclear factor kappa B kinase subunit beta; *IL2*: interleukin 2; *IL2RG*: interleukin 2 receptor subunit gamma; *IL6*: interleukin 6; *IL6R*: interleukin 6 receptor; *IRAK*: interleukin 1 receptor associated kinase; *JAK1*: janus kinase 1; *JAK2*, janus kinase 2; *LALBA*: lactalbumin alpha; *LIFR*: LIF receptor subunit alpha; *LITAF*: LPS induced TNF factor; *LTF*: lactotransferrin; *MCL1*: MCL1 apoptosis regulator, BCL2 family member; *MYC*: MYC proto-oncogene, BHLH transcription factor; *MYD88*: MYD88 innate immune signal transduction adaptor; *NEMO*/*IKBKG*: inhibitor of nuclear factor kappa B kinase regulatory subunit gamma; *NF-κB*: nuclear factor kappa B; *OSMR*: oncostatin M receptor; *P85*: phosphoinositide-3-kinase regulatory subunit 1; *PI3K*: phosphoinositide-3-kinase; *PPP2R3A*: protein phosphatase 2 regulatory subunit B alpha; *PRL*: prolactin; *PRLR*: prolactin receptor; *PTPN6*: protein tyrosine phosphatase non-receptor type 6; *S100A12*: S100 calcium binding protein A12; *STAT3*: signal transducer and activator of transcription 3; *STAT5*: signal transducer and activator of transcription 5; *STING1*: stimulator of interferon response CGAMP interactor 1; *TAK1*/*MAP 3 K7*: mitogen-activated kinase kinase kinase 7; *TIRAP*: TIR domain containing adaptor protein; *TLR2*: toll like receptor 2; *TLR4*: toll like receptor 4; *TNF*: tumor necrosis factor; *TNFRSF17*: TNF receptor superfamily member 17; *TRAF6*: TNF receptor associated factor 6; *WAP*: whey acid protein

The present study provides evidence that sustained hyperprolatinemia at the end of pregnancy, hence in the absence of suckling piglets, sends conflicting pro-survival and cell death signals to MECs (summarized in Fig. 3). Reception of these signals results in a gland that can simultaneously synthesize milk proteins and initiate mammary involution, as demonstrated by the upregulation of genes associated with the activation of JAK1-STAT3 and TLRs-NF-κB signaling pathways. The sustained milk protein synthesis in the absence of suckling piglets indicates that the switch from the activation of STAT5 to STAT3, known to trigger irreversible mammary gland involution, is partially engaged. On the other hand, the activation of a negative feedback loop that can inactivate the JAK2-STAT5 signaling pathway is suggested through the down-regulation of the PRL receptor transcript and up-regulation of genes known to inhibit JAK2-STAT5 trans-activation (*CISH*, *PTPN6*/*SHP1*). Therefore, it seems that the circulating PRL concentrations in T20 gilts were high enough to sustain milk secretion, but insufficient to prevent the initiation of mammary involution in the absence of milk removal.

In this study, the transcriptomic analysis was performed on the mammary parenchymal tissue that is composed of different cell types including epithelial and myoepithelial cells, fibroblasts, adipocytes, endothelial cells and immune cells such, as neutrophils and macrophages, that are also present during the involution process [71]. We must therefore take into account that enriched pathways and genes identified in the present study may originate from different population of cells interacting with each other. Cell type-specific transcriptomic analyses using fluorescence-activated cell sorting, as previously reported by Kramer et al. [73], would allow for better characterization of these interactions.

Although there is no doubt that the process of involution is underway, determination of the exact involution stage in T20 gilts is complex because up-regulated genes known to trigger the lysosomal-mediated cell death (first phase of involution), or to be associated with the apoptotic cell death occurring later on, were both identified. Additional experiments to assess executioner caspases (caspases 3, 6 or 7) activities would certainly help in determining the exact stage of mammary involution. Finally, it remains unclear whether providing domperidone for longer periods of time, in the absence of milk removal, would allow for completion of the whole involution program and remodeling of the gland to its pre-pregnancy state. Additional experiments with longer treatment periods are therefore needed to answer that question.

## Methods

Experimental procedures were conducted according to the current guidelines of the Canadian Council on Animal Care [74] and were approved by the institutional animal care committee of the Sherbrooke Research and Development Centre of Agriculture and Agri-Food Canada.

### Animals and treatments

The present study is part of a larger research project on the effects of sustained hyperprolactinemia on the mammary development of gilts in late pregnancy [6]. A total of fifty-two Yorkshire x Landrace gilts were bred via artificial insemination with pooled semen from Duroc boars of proven fertility [6]. These gilts were provided by the groupe Cérès Inc. (Lévis, Quebec, Canada) and were sent to the swine complex of the Sherbrooke Research and Development Centre (Agriculture and Agri-Food Canada) upon reaching 160–170 days of age. On d 90 of gestation, gilts were randomly assigned to three experimental groups and received daily intramuscular injections (3 mL) of (1) canola oil until d 109 ± 1 of gestation (CTL, *n* = 18); (2) the dopamine receptor antagonist domperidone (0.5 mg/kg of BW) until d 96 ± 1 of gestation (T7, *n* = 17) or; (3) domperidone (0.5 mg/kg BW) until d 109 ± 1 of gestation (T20, *n* = 17). The domperidone (Glentham Life Sciences, Corsham, Wiltshire, UK) was suspended in canola oil twice a week. To rapidly increase circulating PRL concentrations, the T7 and T20 treated gilts were given 100 mg of domperidone by oral administration at 8:00 and 20:00 h, on days 90, 91, 92, and 93 of gestation. Domperidone was delivered in a pellet made of 15 g of finely ground corn and 10 mL of water. Control gilts received the corn pellet alone. Details on housing and diets are provided in Caron et al. [6]. Gilts were slaughtered on d 110 ± 1 of gestation and mammary parenchymal tissue was sampled, immediately frozen in liquid nitrogen and kept at − 80 °C. Gilts were stunned with a captive bolt pistol prior to slaughter by exsanguination to ensure that the animals were killed humanely. This procedure was performed at the Sherbrooke Research and Development Centre by trained professionals following a normalized procedure and according to the current guidelines of the Canadian Council on Animal Care.

### RNA extraction and evaluation of integrity

Total RNA was isolated from 30 mg of parenchymal tissue (*n* = 52) using the RNeasy Mini Kit (Qiagen, Toronto, ON, Canada) and included a DNAse I digestion step directly on columns. The integrity of extracted RNA was determined using the Agilent 2100 Bioanalyzer system (Agilent Technologies, Santa Clara, CA, USA) and its concentration was assessed using the NanoDrop Spectrophotometer ND-1000 (NanoDrop Technologies, Wilmington, DE, USA). The RNA samples used to perform the microarray analysis were randomly selected (*n* = 6 CTL and *n* = 6 T20) and had RNA Integrity Numbers (RIN) ranging from 7.5 to 8.1, thus meeting quality criteria for microarray analyses [75].

### Microarray hybridization and data analyses

Microarray hybridizations were performed at the “Centre d’expertise et de services Génome Québec”, at McGill University (Montreal, QC, Canada). For each sample, the total RNA (100 ng) was reverse transcribed into sense-strand cDNA and probes were biotinylated using the GeneChip® WT Terminal Labeling Kit (Thermo Fisher Scientific, Waltham, MA, USA) according to the manufacturer’s recommendations. The biotinylated probes were hybridized to the Affymetrix GeneChip® Porcine Gene 1.0 ST Array (Thermo Fisher Scientific) providing 394,580 unique 25-mer probes for a total of 19,202 porcine genes. GeneChips were then washed in a GeneChips® Fluidics Station 450 (ThermoFisher) using the GeneChip Hybridization, Wash and Stain kit (ThermoFisher) according to the manufacturer’s instructions. Arrays were scanned using a GeneChip™ Scanner 3000 7G System (Thermo Fischer Scientific) and the Affymetrix Genechip® Command Console software (Thermo Fischer Scientific) was used to produce. CEL file format. Array background adjustment, data normalization and summarization of probe sets were performed with the Affymetrix Expression Console software and using the Robust Multichip Average (RMA) [76] algorithm. The identification of differentially expressed genes (DEG) and clustering analyses was carried out with the Transcriptome Analysis Console software (Thermo Fischer Scientific). A cut-off threshold of 1.5-fold and an adjusted *P*-value < 0.05 was applied for determination of differentially expressed genes between T20 (*n* = 6) and CTL (*n* = 6) gilts. The complete list of identified DEGs is available in Additional file 1: supplementary Table 1.

### Functional enrichment analysis of differentially expressed genes

Functional annotation clustering of DEGs was performed using the Database for Annotation, Visualization and Integrated Discovery (DAVID) [77] bioinformatics resource database (version 6.8, http://david.abcc.ncifcrf.gov/). For some microarray transcripts with a LOC identifier, the official gene symbols were obtained through the National Center for Biotechnology Information (NCBI) of the U.S. National Library of Medicine using the *Sus scrofa* (Sscrofa 11.1) genome assembly (Sscrofa11.1 - susScr11 - Genome - Assembly - NCBI (nih.gov)) [78]. The uploaded gene list (one list) included 283 up- and 364 down-regulated unique gene symbols (Additional file 2: supplementary Table 2). Enriched Gene Ontology (GO) terms for biological process and molecular function were determined using the *Sus scrofa* reference gene list and the GO Direct parameter, that exclude less specific parent terms. The minimum number of genes for enriched GO terms was set at six and the expression analysis systemic explorer (EASE) score threshold was set at 0.05 (modified Fisher Exact *P*-Value). A Benjamini-Hochberg correction was also applied to establish statistical significance [79]. The biological pathways enrichment analysis was performed using the Kyoto Encyclopedia of Genes and Genomes (KEGG) PATHWAY [80] from the DAVID database. The *Sus scrofa* gene annotation list was used as background. Gene count and EASE thresholds were set at six and 0.05, respectively. A Benjamini-Hochberg correction to account for false discovery rate was applied [79].

The STRING database (version 11.0) was used to visualise interconnections among enriched KEGG pathways and genes. This online STRING tool (https://string-db.org/) generates direct (physical) and indirect (functional) protein-protein interaction networks [81]. For these analyses, the confidence score was set at 0.400 (medium confidence) and text mining, experiments, databases, co-expressions, neighborhood, gene fusion and co-occurrence were selected as active interaction sources. The minimum interaction score was set at > 0.400 and KEGG pathway enrichment significance at *P* <  0.05 (FDR Benjamini-Hochberg). Nodes (genes/proteins) devoid of any interaction were excluded from the resulting STRING network.

### Quantitative RT-PCR analyses

Quantitative PCR analyses were performed to validate microarray data and to analyze the expression profile of selected genes in all CTL, T7 and T20-treated gilts (*n* = 52). Twenty-six genes (13 up- and 13 down-regulated) were selected based on their relevance with regards to known or suspected mechanisms regulating development and involution of the mammary gland and also included genes having the highest fold change between T20 and CTL treatments (Table 1). Total RNA was isolated as described above. First strand cDNA was amplified using oligo (dT) 20 primers and the SuperScript™ IV Reverse Transcriptase (Thermo Fisher Scientific), as recommended by the manufacturer. Real-time qPCR analyses were carried out using an ABI 7500 Fast Real-Time PCR System (PE Applied BioSystems, Foster City, CA, USA). The PCR reactions consisted of 5.0 μL of 2x Power SYBRGreen Master Mix (PE Applied BioSystems), 3 μL of diluted cDNA (1/30), optimal primer concentrations (Additional file 2: supplementary Table 3) and 0.05 μL of UNG AmpErase (PE Applied BioSystems) made up to a final reaction volume of 10 μL. Cycling conditions were 2 min at 50 °C to activate AmpErase, 10 min at 95 °C and 40 cycles of 15 s at 95 °C, and annealing and polymerization for 45 s at 60 °C. At the end of amplifications, a melting curve analysis was generated to assess amplified fragments specificity. The PCR reactions were performed in triplicate and standard curves were established in duplicate for each selected and reference (RG) genes. Two RG were amplified: actin beta *(ACTB)* and peptidylprolyl isomerase A (*PPIA*) (Additional file 2: supplementary Table 3). For each gene and RG, a standard curve was generated using serial dilutions of pooled cDNA from parenchymal tissue [82]. The amplification efficiencies (E) of target genes and RG are reported in Additional file 2: supplementary Table 3 and were calculated from the slopes of standard curves using the following equation: E = 10 ^[− 1/slope]^, followed by a conversion of E into a percentage ((E-1) × 100). The relative mRNA abundance of selected genes was then calculated using the relative standard curve method as described by Applied BioSystems [83]. The relative mRNA abundance ratio was obtained by dividing the relative quantity unit of candidate genes by those of RG and mean values from triplicates were used for statistical analyses. Using the NormFinder algorithm [84] from Excel-Tools-Add-ins, the combination of *ACTB* and *PPIA* was identified as the best combination of RG to be used for mRNA abundance normalization.

### Statistical analyses

The relative mRNA abundance data were analyzed using a one-way analysis of variance with heterogeneous variances followed by all-pairwise multiple comparisons with a Tukey correction (Mixed procedure of SAS; SAS Institute Inc. 2002, Cary, NC, USA). A non-parametric Kruskal-Wallis test was performed to confirm the global treatment effect. The model included the effect of treatment, with the residual error being the error term used to test main effects of treatment. LSmeans are presented with their individual SEM. Statistical significance was set at *P* ≤ 0.05 and tendencies at 0.05 < *P* ≤ 0.10.

## Supplementary Information

**Additional file 1: Supplementary Table 1** Complete database of differentially-expressed genes in the mammary parenchymal tissue of T20 vs CTL gilts. The following filter criteria were used: 1.5-fold change and an adjusted *P*-value ≤0.05.

**Additional file 2: Supplementary Table 2.** List of up- and down-regulated genes in the mammary parenchyma of T20 compared to CTL gilts. This is a unique gene list that was uploaded in the DAVID bioinformatics resource database for functional annotation clustering of differentially expressed genes. **Supplementary Table 3.** Primer sequences used for qPCR amplifications of differentially expressed genes in the mammary parenchyma of CTL, T7 and T20 gilts.

## Data Availability

All data generated or analysed during this study are included in this published article and its supplementary information files.

## Abbreviations

CTL: Control
DEGs: Differentially expressed genes
EASE: Expression analysis systemic explorer
ER: Endoplasmic reticulum
FA: Fatty acid
FC: Fold change
FES: Fold enrichment score
GO: Gene Ontology
JAK-STAT: Janus kinase/signal transducer and activator of transcription
LPS: Lipopolysaccharides
MEC: Mammary epithelial cells
NF-κB: Nuclear factor kappa B
PI3K-Akt: Phosphatidylinositol 3-kinase/AKT serine/threonine kinase
PRL: Prolactin
PRLR: Prolactin receptor
RG: Reference gene
T7: Domperidone provided for 7 days (d 90 to 96) to late-pregnant gilts
T20: Domperidone provided for 20 days (d 90 to 109) to late-pregnant gilts
