# Supplementary material for: Effects of sustained hyperprolactinemia in late gestation on the mammary parenchymal tissue transcriptome of gilts
Source: BMC Genomics. 2023 Jan 24;24:40. doi: 10.1186/s12864-023-09136-4 (PMC9875420; doi:10.1186/s12864-023-09136-4)
Supplement: Supplementary file 2 — Additional file 2: Supplementary Table 2. List of up- and down-regulated genes in the mammary parenchyma of T20 compared to CTL gilts. This is a unique gene list that was uploaded in the DAVID bioinformatics resource database for functional annotation clustering of differentially expressed genes. Supplementary Table 3. Primer sequences used for qPCR amplifications of differentially expressed genes in the mammary parenchyma of CTL, T7 and T20 gilts. [file 12864_2023_9136_MOESM2_ESM.docx]

| **Supplementary Table 2.** List of up- and down-regulated genes in the mammary parenchyma of T20 compared to CTL gilts^1^ | | |
| --- | --- | --- |
|  | Count | Official gene symbols |
| Up-regulated genes  (T20 vs CTL) | 283 | ACKR2, ACSL3, ACSL6, ACSS3, ACTG2, ADAMTSL4, ADCY1, AGPAT3, ALDH1B1, ALDH2, AMIGO3, AMPD3, ANGPTL4, ANKH, ANKRD22, ANPEP, ARRDC3, ARSA, ATP1B2, ATP2B2, BCHE, BCO2, BDH1, BHLHE40, BMP1, BRICD5, C3, C9, CAPG, CASP1, CCL23, CCND2, CCR10, CD14, CD209, CD36, CD59, CD69, CD79A, CD82, CEBPD, CHDH, CHI3L2, CHL1, CHST8, CIDEA, CISH, CITED2, CNN1, CP, CPM, CREB3L1, CRELD2, CRISP3, CROT, CSF2RA, CSN1S2, CSN3, CSRP1, CTSC, CTSH, CXCL2, CXCL8, CYP27A1, CYP27B1, CYP4A24, CYP51, CYSTM1, CYTIP, DAB2, DDR2, DERL3, DMRTA1, DNAJB11, DNAJC6, DNASE1L1, DOK5, DPH3, DPP10, DRAM2, DUOX1, DUOXA1, DYNC2LI1, ECM1, EDEM1, EGF, ELOVL5, ELOVL6, EMP1, EZH2, FAM46A, FAM46C, FLVCR2, FOLR1, FOLR2, FOS, FOXO1, FRMPD1, FXYD2, GALNT15, GALNT3, GCLC, GFPT1, GHR, GINM1, GNB4, GPM6B, GPX1, GRAMD1B, GRN, Grp94, GSKIP, HCRTR1, HM13, HP, HS6ST1, HSD17B7, HSPA13, HSPA5, HTATIP2, HYOU1, IFNGR2, IKZF2, IL13RA1, IL1RN, IL2RG, IL6R, ILVBL, IQUB, ITGA2, ITGB6, JCHAIN, KCNA3, KCNJ15, KCNQ1, KCTD8, KDELR3, LALBA, LCLAT1, LGMN, LIPG, LITAF, LOXL1, LPIN2, LRG1, LSS, LTF, MAB21L3, MAN1A1, MAN1A2, MAN1C1, MAN2A1, MANF, MAP2, MASP2, MCL1, MDFIC, MFSD6, MIR769, MOCS1, MSLN, MT-2B, MYD88, MZB1, NAGA, NECAB3, NEDD9, NFE2L1, NFIL3, NIPAL3, NOD1, NPC2, NT5E, ODAM, ORAI1, OSMR, PAM, PAPSS2, PDEA4, PDIA4, PFKFB2, PGLYRP1, PHLDA2, PIGR, PIM1, PKD2, PLB1, PLEKHD1, PPIB, PPM1M, PPP2R3A, PRDX4, PRNP, PRR16, PRSS8, PSEN2, PTGER4, PTPN6, PYCR1, RALGDS, RASEF, RHBDF1, RHOF, RHOU, RIPK3, RND1, RNF125, RRAS, S100A12, SCD, SCRN1, SDF2L1, SEC24A, SELM, SEPT6, SGMS2, SHMT2, SLAMF7, SLC12A4, SLC16A1, SLC1A1, SLC30A3, SLC35A2, SLC35E1, SLC36A1, SLC39A7, SLC4A4, SLC5A6, SLC6A14, SLC6A6, SMIM22, SMPDL3B, SNORA9, SOD2, SPP1, SRM, SRPR, SRPRB, SRXN1, ST3GAL1, ST6GAL1, STARD10, STARD8, STAT3, STOM, STXBP1, SULF2, SURF4, SYNM, SYS1, TBC1D8B, TC2N, TCN1, TCRA, TIMP2, TINAGL1, TLR2, TLR4, TMC4, TMEM104, TMEM173, TMEM214, TMEM38A, TNFRSF17, TNK2, TP23, TP53INP1, TRAF7, TSPAN3, TUBA1C, TWF1, TXNDC5, UBL3, UPK1B, USP2, VAV1, VDR, VMP1, WAP, WBP1L XDH, ZDHHC2, ZNF792 |
| Down-regulated genes  (T20 vs CTL) | 364 | AASDHPPT, ABCA13, ACAT1, ACBD6, ACOT13, ACSF2, ACVR2A, ADAMTS6, ADAMTS9, ADD3, ADSSL1, AFF2, AHDC1, AHR, AIMP1, AKAP11, AKR1B1, ALDH6A1, AMT, AOAH, AOX1, APBB2, ARHGEF4, ATG10, ATIC, ATP6V1D, AUTS2, B3GALT2, B3GALT5, BACE1, BACH2, BEX1, BIRC5, BLZF1, BPHL, BTF3, C10H9orf3, C12H17orf49, C1GALT1, C1H14orf166, C3H2orf40, C9H11orf97, CACNA2D3, CAMK1G, CASK, CAT, CCDC14, CCDC181, CCND1, CCNJ, CCT2, CCT7, CDC5L, CDCA7L, CDS1, CEP290, CGNL1, CHODL, CHRDL2, CHRM1, CHRNB4, CIRBP, CKMT2, CLYBL, CNIH2, COBL, COL9A1, COPG2, CPSF6, CRLS1, CSRP2, CTNNB1, CXCR4, DBI, DGUOK, DLG3, DMD, DNAJC28, DNMT3A, DNMT3B, DSP, DSTYK, DTL, DTNB, DTX4, E4, ECSCR, EEF1B2, EEF1D, EIF2S3, EIF3D, EIF3E, EIF3G, EIF4A2, EIF4EBP2, EMP2, ENAH, EPC2, ERBB4, ERCC8, ESD, ETFB, FAH, FAM135B, FAM13C, FAM171A1, FAM214A, FAM73A, FAP2C, FAR1, FAT2, FBXL7, FCGR2B, FGD1, FHDC1, FMO2, FMOD, FNBP4, FOPNL, FPGS, FTSJ3, FXYD6, FYB, GKAP1, GLTP, GNAT1, GNG2, GPALPP1, GPR137C, GREB1L, GRHL3, GUCY1A3, HACD3, HELLS, HIBCH, HIGD2A, HMGB2, HMGN2, HMGN3, HNRNPA1, HNRNPDL, HPGD, HPRT1, HS3ST1, HSD17B8, HSDL2, HSP90AB1, HSPE1, IFN-ALPHA-9, IGF1R, IL17RC, IMPDH2, IPO5, IRX3, ITPR2, KAT7, KCNH8, KHDRBS1, KIAA0040, KIAA1191, KIF16B, KITLG, KLHL15, KREMEN1, LCTHIO, LDOC1L, LGR6, LIMK2, LINGO1, LIPT1, LSAMP, LSM3, LTA4H, LYPD6B, LZTS1, MALL, MAOB, MAP4K2, MARK4, MB21D2, MBTD1, MEGF8, MID2, MIR181B-1, MIR505, MMP16, MRPL14, MRPL39, MRPL46, MT3, MTERF2, MTMR12, MTMR2, MTMR9, MYB, MYC, MYLIP, MYO5C, N4BP2, NAF1, NAP1L1, NCALD, NEBL, NET1, NFIX, NGFRAP1, NICN1, NIPAL1, NOL4L, NOP16, NPM1, NRAP, NRP1, NSA2, NTRK3, NUDT5, OCRL, ORMDL1, OSGEPL1, P2RX7, PABPC4, PAPLN, PAQR6, PBX2, PCBD1, PCNX, PDE4D, PDE6D, PDE9A, PDGFC, PDLIM1, PDZRN3, PEBP1, PHACTR1, PHC1, PICK1, PIK3C3, PLCH1, PMM1, PPARGC1A, PPP1R3B, PRDX3, PRDX6, PRKRIR, PRLR, PSAT1, PSME1, PSME2, PTK7, RAB11FIP5, RAI2, RANBP17, RAPGEF4, RARG, RASSF4, RAVER2, RBFOX1, RCBTB1, RGS2, RNF113A, RNF144B, RNF217, RNF24, RPL10A, RPL11, RPL22, RPL24, RPL27, RPL31, RPL34, RPL35A, RPL36, RPL5, RPL9, RPS20, RPS3, RPS3A, RSPO1, RUFY1, S100B, SCAI, SCARB2, SCP2, SCRG1, SEC22C, SEC61A2, SEPT10, SERTAD4, SESN3, SFXN4, SFXN5, SHC4, SIMC1, SLC16A10, SLC16A11, SLC25A35, SLC25A36, SLC26A7, SLC44A4, SLC5A5, SMN1, SNRPD1, SNRPE, SNRPF, SNRPG, SNX5, SORCS1, SOX9, SSRP1, ST3GAL5, ST6GAL2, STK26, STOX2, SUB1, SUCLG1, TAF1B, TBC1, TBC1D14, TBC1D22A, TCF7L2, TDRD1, TESC, TET1, TGFA, THRB, TIAM1, TIMM9, TM4SF1, TMEM106A, TMEM141, TMEM164, TMEM63B, TMLHE, TOMM20, TRAP1, TRIM13, TRPV2, TSPAN14, TTC29, TTC38, TTC8, TTC9C, TTLL7, UBR5, UGT8, UNC5B, UXS1, VAMP4, VWA3B, WBP2, WDHD1, WEE1, WISP3, XPNPEP1, ZBTB33, ZCCHC11, ZDHHC17, ZFP62, ZMIZ1, ZNF277, ZNF518A, ZNF567, ZNF618, ZNRF3 |
| ^1^These genes were included in a unique gene list that was uploaded in the DAVID bioinformatics resource database for functional annotation clustering of differentially expressed genes. | | |

| **Supplementary Table 3.** Primer sequences used for qPCR amplifications of differentially expressed genes in the mammary parenchyma of CTL, T7 and T20 gilts | | | | | |
| --- | --- | --- | --- | --- | --- |
| Genes | Primer sequences (5’ 3’) | Genebank  accession No. | Product  size (bp) | Concentration  (nM) | Amplification Efficiency (%)^1^ |
| **Selected up-regulated genes** | | | | | |
| *ACKR2* | (F)TGGCAGAGTCTTTCTGCCTATC (R)AGAGCAAGACCACGAGAAGAAG | NM_001256773 | 86 pb | 300/300 | 97,8 |
| *ANPEP* | (F)TCTCCTTCTCCAACCTCATC  (R)CCCACATCCATGTTGTTCTT | NM_214277 | 100 pb | 150/150 | 101,4 |
| *BHLHE40* | (F)ACCTTGAAGCATGTGAAAGCAC (R)GACATTTCTCCCCGACAGATCA | NM_001245010 | 108 pb | 300/300 | 99,4 |
| *CD14* | (F)GCAGAGGCTTTGAGGACCTTAT (R)CGGACGTCTTCGTCGTCTATTT | NM_001097445 | 125 pb | 300/300 | 102,1 |
| *CD36* | (F)AGCCTCATTTCCACCTTTC (R) AGCTCCAAACACAGCATAG | NM_001044622 | 92 pb | 150/150 | 98,3 |
| *CEBPD* | (F)TTCAGCCGAAAACGAGAAGTTG (R)GGTAGCTGCTTGAAGAATCGC | XM_005663091 | 87 pb | 150/150 | 102,3 |
| *CIDEA* | (F)GCCACCATGTACGAGATGTACT (R)TGCCCATGTAGATGAGACACTG | NM_001112696 | 133 pb | 300/300 | 97,8 |
| *ECM1* | (F)TGTTGCTTCTGTAGTCCCAGAG (R)GCAGCATAGCCAGCTTCTTG | XM_021089905 | 93 pb | 300/300 | 99,5 |
| *EGF* | (F)ACGGTGGTGTGTGTATGTATATTG (R)CCATTTCAAGTCTCTGTGCTGAC | NM_214020 | 101 pb | 300/300 | 98,4 |
| *LALBA* | (F)TCCTGGATGATGACCTTACT (R)TCTGAACAGAGTGCTTTATGG | NM_214360 | 101 pb | 150/150 | 97,8 |
| *LRG1* | (F)TCTTGGAGCCCAGAAGGAA (R)CCTTGGCTGAGACCACAAATAG | XM_003123071 | 98 pb | 300/300 | 97,3 |
| *SPP1* | (F)TCCTAGCGCCACAGAATACTATTTC (R)GCTCAGGGCTTTCGTTGGA | NM_214023 | 90 pb | 150/150 | 96,8 |
| *TC2N* | (F)CCTTCCAAGCTCATCAACA (R)CAGTAACCGTGTCTTCTTCTTA | XM_005656445 | 101 pb | 300/300 | 99,7 |
| **Selected down-regulated genes** | | | | | |
| *CAMK1G* | (F)CTACAGCAAGGCCGTGGATT (R)TCATCCCAGAACGGAGACTCA | NM_001243934 | 150 pb | 300/300 | 103,5 |
| *CHODL* | (F)TGGAGGTCCCTACCTTTAC (R)CGGGAGCTGTTGGATAAAT | XM_003483343 | 99 pb | 300/300 | 97,6 |
| *COL9A1* | (F)GGTGACAGGGGTGTAGTTGG (R)CCCCAGGGTTACCTACGGAT | XM_003121273 | 139 pb | 300/300 | 102,1 |
| *CXCR4* | (F)CATCGGGATCAGCATCGACT (R)CAAGGGCCTCAGTGATGGAA | NM_213773.1 | 104 pb | 300/300 | 101,3 |
| *ERBB4* | (F)ATAGACCCAGAGAAACTGAATG (R)CACCAATAGTCACCAGGTTAG | XM_021075968 | 125 pb | 300/300 | 97,8 |
| *FMO2* | (F)GCTGGAGAAGTCAACCCTTG (R)CTCCGAAGGCAAGGTACATAAG | XM_003130104 | 127 pb | 300/300 | 103,4 |
| *NCALD* | (F)GAAGATGAGTCAACCCCAGAG (R)CAGGCGCACTATGGACG | NM_001244465 | 129 pb | 300/300 | 98,6 |
| *P2RX7* | (F)GACGCTCTGTTCCTCTAAC (R)GGTCTTCTGCTTCCCTTTAT | XM_001926804 | 107 pb | 300/300 | 101,8 |
| *RCBTB1* | (F)GAGGAATCACCGTGGAGAATG (R)CCGCAGTCTGTGTAACTTCTG | XM_013980497 | 126 pb | 300/300 | 97,6 |
| *RGS2* | (F)GGAGAAGATGAAGCGGACCC (R)GGTTTGCTTTTCTTGCCGGT | NM_001044600 | 108 pb | 300/300 | 96,9 |
| *SCRG1* | (F)TGGGATGGGAAGGGATGTGA (R)ATGGTGCTGTCTGGAATACAGG | XM_021072436 | 149 pb | 300/300 | 97,7 |
| *SHC4* | (F)CTGCCCCATACGGTGTGAAA (R)ATGCATCTCGCTGGGACTG | XM_003121520 | 137 pb | 300/300 | 98,6 |
| *TDRD1* | (F)ATAGGGCAACCTTGTTGTGC (R)TTGGAGTTCGTCGGCAATCA | XM_021073713 | 144 pb | 300/300 | 101,3 |
| **Reference genes** | | | | | |
| *ACTB* | (F)CATCACCATCGGCAACGA (R)GGATGTCGACGTCGCACTT | XM_003124280 | 128 pb | 300/300 | 96,2 |
| *PPIA* | (F)GGTCCTGGCATCTTGTCCAT (R)TCATGCCCTCTTTCACTTTGC | NM_214353 | 130 pb | 300/300 | 98,4 |
| Abbreviations: *ACKR2*: atypical chemokine receptor 2; *ACTB*: beta-actin; *ANPEP*: alanyl aminopeptidase, membrane; *BHLHE40*: basic helix-loop-helix family member E40; CAMK1G: calcium/calmodulin dependent protein kinase IG; *CD14* and *36*: cluster of differentiation 14 and 36; *CEBPD*: CCAAT/enhancer binding protein delta; *CHODL*: chondrolectin; *CIDEA*: cell death inducing DFFA like effector A; *COL9A*: collagen type IX alpha 1 chain COL9A1; *CXCR4*: C-X-C motif chemokine receptor 4; *ECM1*: extracellular matrix protein 1; *EGF*: epidermal growth factor; *ERBB4*: erb-b2 receptor tyrosine kinase 4; *FMO2*: flavin containing monooxygenase 2; *LALBA*: lactalbumin alpha; *LRG1*: leucine rich alpha-2-glycoprotein 1; *NCALD*: neurocalcin delta; *PPIA*: Peptidylprolyl isomerase A (Cyclophiline A); *P2RX7*: purinergic receptor; *RCBTB1*: RCC1 and BTB domain containing protein 1; *RGS2*: regulator of G protein signaling 2; *SCRG1*: stimulator of chondrogenesis 1; *SHC4*: SHC adaptor protein 4; *SPP1*: secreted phosphoprotein 1; *TC2N*: tandem C2 domains, nuclear; *TDRD1*: tudor domain containing 1.  ^1^ Amplification efficiency (E) was calculated with E = 10^(-1/slope)^ | | | | | |
